# P wave dispersion and cardiac involvement in patients with juvenile idiopathic arthritis

**DOI:** 10.1186/1546-0096-9-S1-P142

**Published:** 2011-09-14

**Authors:** Bulent Koca, Ozgur Kasapcopur, Suleyman Bakari, Emre Celik, Ayse Guler Eroglu, Levent Saltik, Funda Oztunc, Ozden Calay

## Background and aim

Juvenile idiopathic arthritis (JIA) is the most common rheumatologic disorder of childhood. Cardiac involvement as pericarditis, myocarditis and valvular disease is common in JIA (JIA). There are, however, few descriptions concerning systolic and diastolic functions of the left ventricle (LV) in children with JIA. P wave dispersion (PWD) is a sign for the prediction of atrial fibrillation (AF). A recent study found that rheumatoid arthritis patients had an abnormally high P wave duration and PWD , markers for supraventricular arrhythmogenicity. The study was to assess PWD and its relation with systolic and diastolic function of the LV in a group of children with JIA.

## Methods

We performed electrocardiography and Doppler echocardiography on 50 patients with JIA and 70 healthy controls. Maximum and minimum P wave duration were obtained from electrocardiographic measurements. PWD defined as the difference between maximum and minimum P wave duration was also calculated.

## Results

No statistically significant differences were found between the groups in minimal, maximal P wave duration and PWD (Table 1). Among the diastolic parameters, increased late flow velocity, decreased early flow velocity and prolonged isovolumic relaxation time reflected an abnormal relaxation form of diastolic dysfunction. During 12 months of follow-up, no supraventricular arrhythmias were documented in either group.

**Table 1:** Electrocardiographic measurements of the JIA patients and controls

	JIA (mean ± SD)	Controls (mean ± SD)	P value
Maximum P wave duration (ms)	79.62 ± 11	81.31 ± 9.11	NS
Minimum P wave duration (ms)	55.20 ± 11.12	56.63 ±10.41	NS
P wave dispersion (ms)	24.42 ± 11.40	25.62± 10.24	NS

## Conclusions

P wave duration and PWD was not found to be higher in JIA patients than healthy control subjects. Larger observational studies and prolonged follow-up are therefore required before definitive conclusions can be made.

